# Basal Cell Carcinoma Arising Within a Bacillus Calmette–Guérin Vaccination Scar With Prolonged Inflammatory Response: Case Report

**DOI:** 10.1002/ccr3.70275

**Published:** 2025-03-05

**Authors:** Marwa Hallal, Zeina Tannous

**Affiliations:** ^1^ Gilbert and Rose‐Marie Chagoury School of Medicine Lebanese American University Beirut Lebanon; ^2^ Department of Dermatology, Gilbert and Rose‐Marie Chagoury School of Medicine Lebanese American University Beirut Lebanon; ^3^ Department of Dermatology, Wellman Center for Photomedicine Massachusetts General Hospital, Harvard Medical School Boston Massachusetts USA

**Keywords:** basal cell carcinoma, BCG vaccination, case report, scar

## Abstract

Infiltrative basal cell carcinoma (iBCC) can rarely arise within scar tissue, as demonstrated by this unique case of BCC developing in a Bacille Calmette–Guérin (BCG) vaccination scar. This underscores the importance of monitoring prolonged post‐vaccination inflammation and exploring its potential link to skin cancer development.

## 1 Introduction

Basal Cell Carcinoma is the most common form of skin cancer, accounting for approximately 80% of all nonmelanoma skin cancers worldwide. It typically arises from the basal cells of the epidermis and is strongly associated with prolonged ultraviolet (UV) exposure leading to DNA damage and mutations in genes such as PTCH1 and p53. Epidemiologically, basal cell carcinoma predominantly affects Caucasians, with a higher incidence in older adults and those with a history of extensive sun exposure [1].

Despite the relatively low mortality rate of BCC compared to other forms of skin cancer, its rising incidence presents a public health challenge. Various risk factors, such as prolonged sun exposure, immunosuppression, and genetic predispositions, all play a role in its development. While BCC predominantly arises in sun‐exposed areas of the skin, atypical presentations can occur, particularly in individuals with a history of skin cancer or other predisposing factors [1].

The development of BCC within a scar, including those resulting from Bacille Calmette–Guérin (BCG) vaccination, is a rare phenomenon with few cases reported in the literature [2, 3, 4, 5, 6, 7, 8, 9]. We present a rare case of infiltrative basal cell carcinoma (iBCC) arising more than 50 years after BCG vaccination within the corresponding scar. The uniqueness of this case lies in the prolonged inflammation at the vaccination site, which may suggest a potential association between a chronic inflammatory response and BCC development.

## 2 Case History

A 62‐year‐old male, with Fitzpatrick skin type III, presented with an erythematous, scaly patch located at the site of his childhood Bacillus Calmette‐Guérin (BCG) vaccination scar on the right upper arm. He had received the BCG vaccine in 1970, prior to traveling to Jordan, and experienced a significant local reaction, including prolonged swelling at the injection site that persisted for several weeks. He had no history of skin cancer or childhood burns, and no family history of the disease.

**FIGURE 1:**
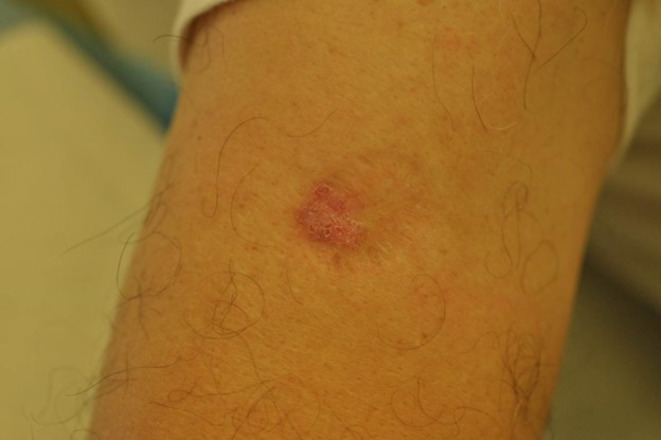
Erythematous scaly patch measuring 22.0 mm × 17.0 mm, located within an atrophic scar on the deltoid region of the patient's right upper arm.

Three to four months prior to his presentation, the patient noticed progressive enlargement of the scar, which became increasingly erythematous and scaly, though he reported no associated symptoms. On examination, a 22 mm × 17 mm erythematous, scaly plaque was observed within an atrophic scar on the deltoid region of the patient's right arm (Figure 1).

## 3 Methods

**FIGURE 2:**
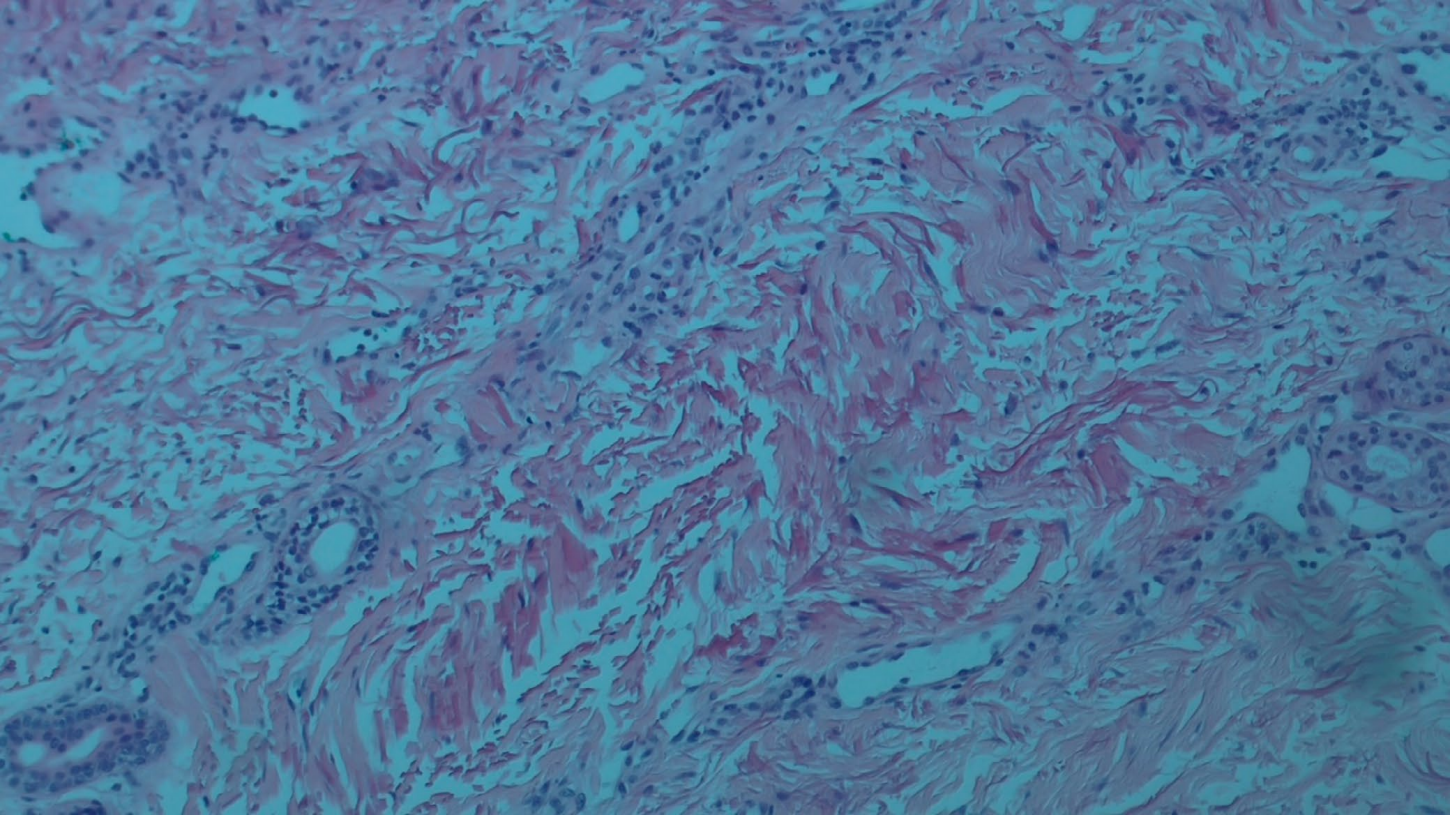
Narrow strands of atypical basaloid cells infiltrating desmoplastic stroma, with scattered inflammation and collagenous scar tissue, characteristic of infiltrative basal cell carcinoma [H&E, 60×].

A punch biopsy revealed narrow, elongated strands of atypical basaloid cells infiltrating the dense, desmoplastic stroma beneath the epidermis. The tumor cells exhibited hyperchromatic nuclei, scant cytoplasm, and peripheral palisading, though less prominent compared to other BCC subtypes. The infiltrative growth pattern is characteristic of infiltrative basal cell carcinoma (iBCC). This was accompanied by scattered inflammatory infiltrates, retraction spaces, and underlying collagenous scar tissue, consistent with the site of a prior vaccination scar. No evidence of vascular or perineural invasion was noted (Figure 2).

## 4 Conclusions and Results

Given the lesion's size and the aggressive nature of the infiltrative histopathological subtype, Mohs micrographic surgery was performed, requiring two stages to achieve clear margins and complete excision.

## 5 Discussion

While UV exposure is the primary risk factor for basal cell carcinoma (BCC), other factors include genetic predispositions and environmental exposures [1]. The development of BCC within a scar, including those resulting from BCG vaccination, is a rare phenomenon with only nine cases reported in the literature [2, 3, 4, 5, 6, 7, 8, 9]. The BCG vaccine, primarily used for tuberculosis prevention, can cause localized skin reactions and scarring, which may vary based on the individual's skin type and vaccination technique [8]. A table by Tyczyńska et al. 2022 summarizes previously reported cases of BCC arising in Bacillus Calmette–Guérin (BCG) vaccination scars [8]. The patients ranged in age from 31 to 62 years, with both males and females affected. Lesions presented as ulcerated, scaly, pigmented, or sharply demarcated nodules, with the time from vaccination to lesion onset varying from 1.5 to 52 years. Recent studies indicate that approximately 0.5% of basal cell carcinomas arise from scar tissue, including those associated with trauma, tattoos, leishmaniasis, burns, and surgical scars [3, 10], with an even smaller fraction occurring in post‐vaccination scars [4]. While the precise link between scarring and carcinogenesis remains unknown, no current evidence suggests that vaccination itself causes localized immunosuppression [10].

In this patient, significant local reactions were observed following the BCG vaccination, including prolonged swelling at the injection site lasting several weeks. Given his negative personal and family history of skin cancer and the absence of childhood burns, these findings highlight the potential role of chronic skin inflammation from the BCG vaccination as a trigger for cancer development. Chronic inflammation and persistent irritation can contribute to carcinogenesis by creating a microenvironment rich in cytokines and growth factors, disrupting normal tissue repair and promoting malignant transformation [8, 10].

The emergence of BCC within a BCG scar, as detailed in this case, underscores the rarity of such an occurrence. Nevertheless, it emphasizes the importance of remaining vigilant for potential malignancies irrespective of perceived risk factors. Investigating changes in post‐vaccination scars could facilitate earlier detection and improve patient outcomes. Further research is needed to explore the potential link between prolonged inflammatory responses and BCC development in scar tissue.

## Author Contributions

**Marwa Hallal:** writing – original draft, writing – review and editing. **Zeina Tannous:** conceptualization, investigation, supervision.

## Ethics Statement

This study protocol was reviewed, and the need for approval was waived by The Lebanese American University (LAU) Institutional Review Board (IRB).

## Consent

Written informed consent was obtained from the patient for publication of the details of their medical case and any accompanying images.

## Conflicts of Interest

The authors declare no conflicts of interest.

## Data Availability

All data generated or analyzed during this study are included in this published article.
